# Valproic acid-induced hyperammonemic encephalopathy – a potentially fatal adverse drug reaction

**DOI:** 10.1186/2193-1801-2-13

**Published:** 2013-01-15

**Authors:** Carla Sousa

**Affiliations:** Pharmacy, Hospital de Faro, EPE, Rua Leao Penedo, Faro, 8000-386 Portugal

## Abstract

**Background:**

A patient with an early diagnosed epilepsy Valproic acid is one of the most widely used antiepileptic drugs. Hyperammonemic encephalopathy is a rare, but potentially fatal, adverse drug reaction to valproic acid.

**Case description:**

A patient with an early diagnosed epilepsy, treated with valproic acid, experienced an altered mental state after 10 days of treatment. Valproic acid serum levels were within limits, hepatic function tests were normal but ammonia levels were above the normal range.

Valproic acid was stopped and the hyperammonemic encephalopathy was treated with lactulose 15 ml twice daily, metronidazole 250 mg four times daily and L-carnitine 1 g twice daily.

**Discussion and evaluation:**

Monitoring liver function and ammonia levels should be recommended in patients taking valproic acid. The constraints of the pharmaceutical market had to be taken into consideration and limited the pharmacological options for this patient's treatment.

**Conclusions:**

Idiosyncratic symptomatic hyperammonemic encephalopathy is completely reversible, but can induce coma and even death, if not timely detected.

Clinical pharmacists can help detecting adverse drug reactions and provide evidence based information for the treatment.

## Background

Valproic acid (VPA) has been used in clinical practice since the 60’s, with a relatively favourable safety and efficacy profile. Pancreatitis, hepatotoxicity and teratogenicity are the most significant adverse drug reactions. Cases of hyperammonemia are rarely reported as VPA-induced, probably because this increased level of ammonia in blood can vary between asymptomatic, with an incidence of 16-52% (Carr & Sherwsbury 2007), and clinically relevant levels. Symptomatology due to VPA-induced hyperammonemia include: lethargy, impaired consciousness, focal neurological signs and symptoms and increased seizure frequency. More rare described symptoms are: aggression, ataxia, asterixis, vomiting and coma (Carr & Sherwsbury 2007; Labuzetta et al. 2010).

There is no consensus on whether this symptomatology has any relationship with plasma concentration of VPA. Several studies showed that hyperammonenia may occur with both therapeutic and supratherapeutic concentrations of VPA, indicating that other factors may influence the onset of hyperammonemia. Some authors suggest that symptoms of encephalopathy may develop in patients that have been taking VPA previously, without complications (Carr & Sherwsbury 2007).

A case of encephalopathy due to hyperammonaemia associated with VPA in a patient with therapeutic drug levels and normal liver function is presented.

## Case description

A 60-year-old man, with history of hypertension, diabetes *mellitus*, and alcoholism (in recovery), is admitted because of sudden episode of altered state of consciousness preceded by involuntary movements of the left upper limb, lasting less than five minutes, without motor asymmetries or sphincter incontinence. Neurological examination was normal, except for slowness of speech and difficulty in fixing attention. Vascular study revealed no significant changes and the electroencephalogram (EEG) showed no paroxysmal activity. Considering these results, the presumptive diagnosis of focal cryptogenic epilepsy was given. Patient was treated and discharged with VPA extended release 500 mg twice daily, esomeprazol 40 mg/day and insulin.

Three days after discharge (10^th^ day of valproic acid), the patient was readmitted because of disorientation, speech slowness, somnolence and hemifacial myoclonus. Adherence to treatment and no use of alcohol were confirmed by family members.

Symptoms were interpreted as uncontrolled epilepsy due to quantitative inefficacy (presumptive prolonged post-ictal state), and two days after VPA dosage was augmented to 1000 mg twice daily. Symptoms worsened.

Medical tests revealead blood dyscrasias, glicemia 330 mg/dL and normal liver function. Serum electrolytes, creatinine, blood urea nitrogen and bilirubin were also normal. Serum concentration of total valproic acid was therapeutic (75 mg/L, therapeutic range: 50–100 mg/L).

Idiosyncratic possibilities were considered. Venous serum ammonia level was determined ranging 62,1 μmol/L (normal reference: 5–32 μmol/L) and a repeat EEG showed a generalized slowing with frequent frontocentral diphasic sharp waves, suggesting a metabolic encephalopathy. VPA was stopped and the clinical pharmacist was consulted for evidence based information. A search strategy formulation for Medline/Pubmed was applied: “valproic acid/toxicity” [Mesh]; “Hyperammonemia” [Mesh]. A National Medicines Information Centre was also consulted. Reviews and case reports were used. Critical Appraisal Skills Program was applied for critical analysis of literature (available in: http://www.phru.nhs.uk/casp/critical_appraisal_tools.htm).

Treatment was initiated with lactulose 15 ml twice daily (and adjusted to achieve two-three soft stools/day), L-carnitine 1 g twice daily, metronidazol 250 mg four times daily, complex B vitamins and levetiracetam 500 mg twice daily. Patient improved his consciousness state within 24 hours. Past 11 days ammonia level was 7,1 μmol/L and his level of consciousness resolved. Adjustment in diabetes therapy was made and he was discharged after 2 more days.

## Discussion and evaluation

Symptomatic hyperammonemia due to VPA is quite rare, but can be fatal (Carr & Sherwsbury 2007). In the clinical case presented, the patient experienced decreased level of consciousness, confusion, disorientation and slowness of speech. The hemifacial myoclonus that occurred in three occasions was considered as seizures in a context of uncontrolled epilepsy.

Pharmacological causes of the hyperammonemia induced by VPA have not yet been fully clarified. Studies suggest a hepatic and renal role: amino acids are degraded in the liver to produce ammonia (Cash et al. 2010). Ammonia is both excreted in the urine and converted in urea, which is the major mechanism of nitrogen excretion. Urea is produced in the liver and then transported to the kidneys where it is excreted in the urine or via the intestines where is converted in carbon dioxide and ammonia by the urease-producing bacteria (Labuzetta et al. 2010). Any change in this process may compromise the urea cycle and induce hyperammonemia. In the kidney, VPA affects the renal uptake of glutamine increasing the ammonia production (Carr & Sherwsbury 2007; Cuturic & Abramson 2005). Deficiency of L-carnitine may also decrease urea formation and increase ammonia accumulation in the liver (Cash et al. 2010).

Ammonia levels in the brain are normally two times higher than in the arterial blood, and in case of an acute rise of blood ammonia this ratio goes up, amplifying the toxicity of ammonia. The increased level of ammonia in the central nervous system leads to a higher production and accumulation of glutamine within the astrocytes, causing increased intracellular osmolarity, cerebral oedema and astrocyte dysfunction (Carr & Sherwsbury 2007; Cash et al. 2010).

Although treatment with VPA is generally associated with asymptomatic hyperammonemia there are possible risk factors that can induce and worsen symptomatology: urea cycle disorders, poor nutrition or catabolic state, L-carnitine deficiency, infancy (immature liver function), polymedication, especially other antiepileptic and psychotropic substances. Some well known drug associations that act as risk factors are VPA with: phenytoin; phenobarbital; carbamazepine; topiramate; clozapine and risperidone (Carr & Sherwsbury 2007). In most reported cases patients have concomitant risk medication, and symptoms develop within days or weeks with abnormal liver function (Yee et al. 2005). In this case, patient was taking no medication reported as potential risk factor and hepatic tests were normal. The analysis with the Naranjo probability scale revealed that the adverse drug reaction was probably caused by VPA (Naranjo et al. 1981).

No formal protocol exists for managing these clinical situations, but it is consensual that the first procedure must be the discontinuation of VPA. Usually the pharmacological treatment applied is the same as for hepatic encephalopathy, combined with drugs that may interact with the mechanism of action of VPA (L-carnitine) (Carr & Sherwsbury 2007; Stewart 2008).

Nonabsorbable disaccharides are considered the standard of care for hepatic encephalopathy and include lactitol and lactulose, the latter being the most frequently used (Cash et al. 2010; Naranjo et al. 1981). Lactulose is metabolized into acetic and lactic acids, resulting in acidification of the gastrointestinal lumen. This acidification inhibits the production of ammonia by the coliform bacteria in the intestine. Usually the dosage applied in these cases is 30 ml of lactulose 2 to 4 times a day, adjusting to achieve 2 to 4 soft stools a day and patient compliance (Shiano 2010).

Because we also want to decrease the production of ammonia in the intestine, antibiotics directed to ammonia-producing bacteria can be used. Neomycin, rifaximin and metronidazol have been used (Shiano 2010). Rifaximin has good efficacy with less bacterial resistance and adverse effects than the other antibiotics, and thereby probably more cost-effective (Shiano 2010; Neff 2010).

For this patient, besides treatment with lactulose, the option of using rifaximin and neomycin had to be discarded because rifaximin is not included in our hospital formulary and neomycin does not exist on the national market and has to be imported. For this reason we used metronidazole. Vitamin B complex supplement was added because of previous habits of alcohol intake and levetiracetam was chosen for its profile of safety and efficacy on focal epilepsy. Literature evidence and availability of medicines on the Portuguese market were considered when deciding the treatment options.

## Conclusion

Idiosyncratic hyperammonemic encephalopathy without liver failure is rare, completely reversible, but one of the most severe, potentially fatal, adverse drug reactions to VPA. Intermittent confusional episodes due to hyperammonemia can be easily mistaken with partial seizures inducing medication error, prompting the physician to increase the dose of VPA and thus worsening the hyperammonemia. Recommendations to monitor both liver function and serum ammonia must be considered in patients taking VPA to assist in the early detection of adverse effects. Clinical pharmacists can play an important role in this area, recommending the best treatment available, considering the constraints of the pharmaceutical market, and providing patient follow-up, with evidence based information.
